# HPO-Shuffle: an associated gene prioritization strategy and its application in drug repurposing for the treatment of canine epilepsy

**DOI:** 10.1042/BSR20191247

**Published:** 2019-09-06

**Authors:** Shuguang Wang, Xiangyu Meng, Yuxing Wang, Yemao Liu, Jingbo Xia

**Affiliations:** 1 Hubei Key Lab of Agricultural Bioinformatics, College of Informatics, Huazhong Agricultural University, Hubei Province, P.R. China; 2 Department of Urology, Zhongnan Hospital of Wuhan University, P.R. China

**Keywords:** Canine Epilepsy, Drug Repurposing, Human Phenotype Ontology, Omics Data, Re-ranking

## Abstract

Epilepsy is a common neurological disorder that affects mammalian species including human beings and dogs. In order to discover novel drugs for the treatment of canine epilepsy, multiomics data were analyzed to identify epilepsy associated genes. In this research, the original ranking of associated genes was obtained by two high-throughput bioinformatics experiments: Genome Wide Association Study (GWAS) and microarray analysis. The association ranking of genes was enhanced by a re-ranking system, HPO-Shuffle, which integrated information from GWAS, microarray analysis and Human Phenotype Ontology database for further downstream analysis. After applying HPO-Shuffle, the association ranking of epilepsy genes were improved. Afterward, a weighted gene coexpression network analysis (WGCNA) led to a set of gene modules, which hinted a clear relevance between the high ranked genes and the target disease. Finally, WGCNA and connectivity map (CMap) analysis were performed on the integrated dataset to discover a potential drug list, in which a well-known anticonvulsant *phensuximide* was included.

## Introduction

Epilepsy is a spectrum of neurological disorders characterized by epileptic seizures. It brings considerable burden of disease, affecting nearly 50 million people and 0.5–5% of the canine population [1]. Being classified as idiopathic and secondary epilepsy, 75% of epilepsy is idiopathic without a clearly defined cause, and 25% of it is secondary to abnormalities of the central nervous system (CNS) such as brain injury, cerebral vascular disease and brain tumors [2]. To date, epilepsy treatment mainly relies on medication with anticonvulsant drugs that include phenytoin, carbamazepine and valproate. Although over half of epileptic cases respond to a single-agent or combined antiepileptic treatment, certain subjects show refractory seizures and/or relapse, and suboptimally controlled epilepsy brings severe damage to both physical and mental health. Furthermore, anticonvulsant treatment generally requires long-term administration, and it brings major adverse effects that reduce quality of life. In view of these, development of more effective and less toxic antiepilepsy drugs is a task of great importance.

In terms of treatment for canine epilepsy, certain antiepileptic drugs effective in human beings, including phenytoin, valproic acid and carbamazepine, may not work the same in dogs due to species differnece in metabolism [3,4]. On the other hand, drugs used for canine epilepsy are also associated with adverse effects. For example, Phenobarbital, a widely used anticonvulsant for dogs, may cause adverse reactions in over 50% of dogs, as highlighted in a recent systematic review concerning the safety of antiepileptics in dogs. In addition, Phenobarbital was suspected of having caused pseudo-lymphoma in a dog [5]. Thus, the discovery of novel potential canine antiepileptic drugs is meaningful for canine epilepsy medication.

Traditional drug development is time-consuming and costly [6]. To cope with the urgent needs for new drugs, investigators have paid attention to making use of existing drugs for extended indications. For example, aspirin, an old non-steroid anti-inflammation drug, initially used for pain relief, has shown significant prophylactic activity against cardiovascular diseases and malignancies [7]. Thalidomide, a notorious deformity causing antiemetics, has been successfully redirected for the treatment of autoimmune diseases and hematological malignancies. These examples supported the idea of drug repurposing by discovering new potentials of existed pharmaceutical compounds, which is to ignificantly reduce the time and cost in drug development. A literature-based typical model of drug repurposing is to find indirect relationship among entities in biological literature and unveil new drug–disease pairs, which underlies the idea of the ABC model by Swanson, a powerful paradigm to infer hidden relationships from existing knowledge [8]. By using this model, Swanson searched literature and found hidden clinical usage of fish oil for Raynaud’s syndrome [8]. Another important strategy for drug repositioning is to link drugs with diseases via genes, by integration of gene–drug and gene–disease pairs, which can be established relations either proved by collecting evidences from literature or predicted by datamining the bioinformatics datasets, especially the high-throughput ones on whole genome scale, i.e. Omics data.

Currently, although research efforts have been made to identify key genes associated with epilepsy, the results are still far from unveiling the whole mechanism [9]. For example, the genome-wide *in silico* analysis was regarded as a promising method to unveil hidden facts [10], but unfortunately, the first Genome-Wide Association Study (GWAS) on focal epilepsy carried out on 3445 patients and 6935 controls from Northern Europe found no significant genetic markers as none of the single-nucleotide polymorphisms (SNP) corresponded to a *P*-value that reached Bonferroni adjusted significance [11]. Meanwhile, the second GWAS research covered over 1000 patients from China and identified only two highly correlated variants in the CAMSAP1L1 (CAMSAP1 Like 1) gene, which is a calmodulin-regulated spectrin-associated protein, and it is expressed in neurons and astrocytes in the mammalian nervous system [12]. Till now, though each GWAS work has offered a list of potential candidate genes, few of them has been proven in the wet lab. Since epilepsy is a complex disorder that involves alterations on multiple genes and interactions with external environmental factors [13], this makes it difficult and less likely to find genuine associations with single GWAS analysis. To address this issue, integrated data-mining strategy taking into account either several datasets of a single type or multiple cross-omics datasets can be considered [14,15].

In the present study, we proposed a novel computational pipeline, HPO-Shuffle, which integrated multi-Omics data mining and human phenotype ontology, and showed effectiveness for the identification of compounds that could be repurposed for canine epilepsy treatment, as a preliminary case study. A joint analysis of genome-wide SNPs, transcriptomic gene expression profiling (GEP) and human phenotype ontology (HPO) constitutes the core of our proposed pipeline.

## Materials and methods

### Data and materials

#### Datasets of GWAS, GEP and epilepsy related gene sets

The two previous GWAS studies concerning epilepsy in human beings did not offer data availability and made the replicate experiment and gene ranking attempts impracticable. For the investigation of association between germline genetic variants and canine epilepsy, we used the dataset published with a canine epilepsy GWAS study [16]. The dataset consisted of 4224 gigantic dog blood samples genotyped with a semicustomized SNP array dimensioned up to 180,000. The dogs were examined for multiple complex traits, including 7 common across-breed phenotypes (canine hip dysplasia, elbow dysplasia, cranial cruciate ligament disease, mast cell tumor, lymphoma, portosystemic vascular anomalies and mitral valve degeneration) and 5 common within-breed phenotypes (idiopathic epilepsy in Irish Wolfhounds, granulomatous colitis in Boxers and Bulldogs, lymphoma in Golden Retrievers, mast cell tumor in Labrador Re-trievers and portosystemic vascular anomalies in Yorkshire Terriers). To avoid potential bias introduced by across-breed heterogeneity, we limited our study on idiopathic epilepsy (IE) in Irish Wolfhounds, which consisted of 202 subjects (34 cases and 168 controls) genotyped for 160,727 SNPs. Case dogs were diagnosed by metabolic screening, magnetic resonance imaging and electroencephalogram; while control dogs were >5 years old without history of seizures.

GEP data were collected from the Gene Expression Omnibus (GEO) database, where GSE7486, dataset of a study on human idiopathic epilepsy (https://www.ncbi.nlm.nih.gov/geo/query/acc.cgi?acc=GSE7486, [17]), was selected for the GEP analysis. Under a monozygotic (MZ) twin design, the GSE7486 dataset offered genome-wide gene expression data in lymphoblastoid cell lines of 9 affected MZ twin pairs (case) and 5 unaffected MZ twin pairs (control).

In addition, two epilepsy related gene sets were collected manually, i.e. Relevant Set A through database searching and Key Set B through literature searching.

- Relevant Set A: A wide search of eight databases led to a medium-size epilepsy related gene set, which included 1460 suspected epilepsy related genes. The eight databases were the following: Genetic Association Database (GAD [18], https://geneticassociationdb.nih.gov/), Online Mendelian Inheritance in Man (OMIM [19], http://omim.org/), Clinvar [20] (http://www.ncbi.nlm.nih.gov/clinvar/), Orphanet citeweinreich2008orphanet (http://www.orpha.net/consor/cgi-bin/index.php) [21], DisGeNET [22] (http://www.disgenet.org/web/DisGeNET/menu/rdf), INtegrated TaRget gEne PredItion (INTREPID [23]), GWASdb [24] (http://jjwanglab.org/gwasdb) and the Human Gene Mutation Database (HGMD [25], http://www.hgmd.cf.ac.uk/ac/index.php).
- Key Set B: For the sake of effective evaluation, eight established epilepsy risk genes were collected through manual curation, i.e. EPM2B [26], CTSD [27], TPP1 [28], PPT1 [29], ARSG [30], CLN6 [31], ATP13A2 [32] and LGI2 [33].

#### Ranked gene lists by GWAS and GEP analysis

The raw GWAS data were preprocessed by PLINK v1.9 [34,35] (http://pngu.mgh.harvard.edu/purcell/plink/). Only SNPs with a minor allele frequency (MAF) *>* 0.05 and Hardy–Weinberg Equilibrium (HWE) test *P <* 0.001 were included. A total of 89 085 SNPs passed the initial filtering and were considered in subsequent analyses. A ranked gene list by degree of association quantified by Logistic regression *P* values was obtained.

For GEP analysis, patients with idiopathic absence epilepsy (4 monozygotic twin pairs) and 12 control subjects free of epilepsy (6 monozygotic twin pairs) were selected as the case/control. Background correction, normalization and expression calculation to the raw .cel files were conducted using the RMA algorithm. Transcriptomic matrix of the samples was reconstructed and subjected to linear model based differential expression (DE) analysis with empirical Bayes moderation of the standard errors, through which a ranked gene list by degree of association quantified by Benjamini–Hochberg adjusted Student’s *t*-test *P* values was obtained. The software used for GEP dataset manipulation and analyses was R version 3.4.3. Core packages included the affy (version 1.56.0 [36]) for data collection and preprocessing, and limma (version 3.34.9 [37]) for gene expression matrix construction.

### HPO-Shuffle, re-ranking algorithm for GWAS and GEP gene sets

Designed to rerank GWAS and GEP gene sets and obtain reliable prioritization of epilepsy associated genes, HPO-Shuffle is a reranking algorithm based on the ontology enrichment with respect to the phenotypic ontology system, HPO [38]. HPO is an integrated ontology database that incorporates a well-structured and clearly defined set of 10,088 class terms and 13,326 subclass terms describing human phenotypic abnormalitie. Compatibility of HPO to the Online Mendelian Inheritance in Man (OMIM) and Orphanet databases makes it possible to link all epileptic disorders to relevant abnormalities/phenotypes. Moreover, pair links among HPO terms, diseases and genes are widely available in this database [38].

By querying the OMIM database, 173 disease terms related to epilepsy were retrieved. After mapping them to HPO, 585 human phenotype terms were extracted, based on which was built a specific human phenotype repository (HPR) for later use. The format of the data was shown in Table 1, while the full data were shown in Supplementary Table S1. Epilepsy diseases and related HPO terms*.*

**Table 1:** Sample list of epileptic disorders and HPO terms

OMIM ID	Disease name	HP ID	HP terms in HP repository (HPR)
608217	EPILEPSY, BENIGN NEONATAL, 3	HP:0000006	Autosomal dominant inheritance
		HP:0002069	Generalized tonic-clonic seizures
		HP:0003593	Infantile onset
611277	GENERALIZED EPILEPSY WITH FEBRILE	HP:0003828	Variable expressivity
	SEIZURES PLUS, TYPE 3	HP:0007359	Focal seizures
		HP:0000006	Autosomal dominant inheritance
		HP:0002069	Generalized tonic-clonic seizures
		HP:0002121	Absence seizures
		HP:0010819	Atonic seizures
		HP:0002373	Febrile seizures
267740	RETINAL DEGENERATION AND EPILEPSY	HP:0000007	Autosomal recessive inheritance
		HP:0000546	Retinal degeneration
		HP:0001250	Seizures

Taking advantage of relevance information of the gene and HPO terms, and using HPO terms matching and enrichment, we developed a computational algorithm to filter and rerank candidate genes in the lists obtained from GWAS and DE analyses.

For each gene in GWAS or DE gene list, denote the *P* value for association as *P*(Gene). The smaller this value, the more associated the epilepsy was with the gene in terms of single-nucleotide variation or RNA transcription. For a given gene, Gene_A_, query of its corresponding abnormality/HP term was carried out in HPO database and related HPO terms were collected in the set HPO_Gene_A__. If HPO_Gene_A__ were enriched in the HPR repository, then Gene_A_ was considered more relevant to epilepsy. Then, to update the *P*(Gene_A_), a new metric called HPO-associated value HA(Gene_A_) was calculated as:

(1) $$H\;A({\mathrm{Gene}}_{A})=\frac{P({\mathrm{Gene}}_{A})}{\#(H\;{\mathrm{PO}}_{{\mathrm{Gene}}_{A}})+1}$$

Here, the #(·) operator was to count the number of HPO terms in the set. The ”plus 1” computation was taken to avoid zero valuing. Typically, HA(Gene_A_) = *P*(Gene_A_) if there was no relevant HPO terms for the given gene. The ranked gene lists were updated with the HA values, the smaller ones indicating stronger associations.

### A drug repurposing stragety

#### Integration of HPO-Shuffle with gene clustering and drug filtering tools

Two widely used bioinformatics tools were integrated with HPO-Shuffle. Here, the Weighted Gene Co-expression Network Analysis (WGCNA) was used to cluster modular gene set by using microarray data, while the Connectivity Map [39,40] (CMap) analysis was applied to investigate the key relevance between drugs and modular gene sets.

- As a popular microarray data analysis tool, WGCNA is capable of exploring microarray data by measuring pair-wise correlations between gene expression profiles. WGCNA starts from the level of thousands of genes, identifies clinically interesting gene modules and finally uses intramodular connectivity, gene significance to identify key genes in the disease pathways for further validation. Instead of relating thousands of genes to a microarray sample trait, it focuses on the relation between a few modules and the sample trait. Modules are constructed from the expression data by using hierarchical clustering. By relating modules to specific biological pathways, the focus on these intramodular hub genes amounts to a biologically motivated data reduction and function interpretation.
- Connectivity map (CMap) comprises a database and associated software that is produced by the Broad Institute and is composed of whole-genome gene expression profiles derived from human cell lines that were treated with more than a thousand small molecules [39,40]. CMap performs a difference analysis upon two gene sets that are up-regulated and down-regulated separately in a given condition, and searches and identifies significant molecules that are associated with these gene expression pattern changes.

#### Pipeline of the proposed strategy

A newly developed drug repurposing strategy is proposed in this work, which was implemented for the identification of potential new drugs for canine epilepsy. The aim of this strategy is to make use of HPO-Shuffle, WGCNA and CMap to filter drugs under trial for human to cure dogs.

**Figure 1:**
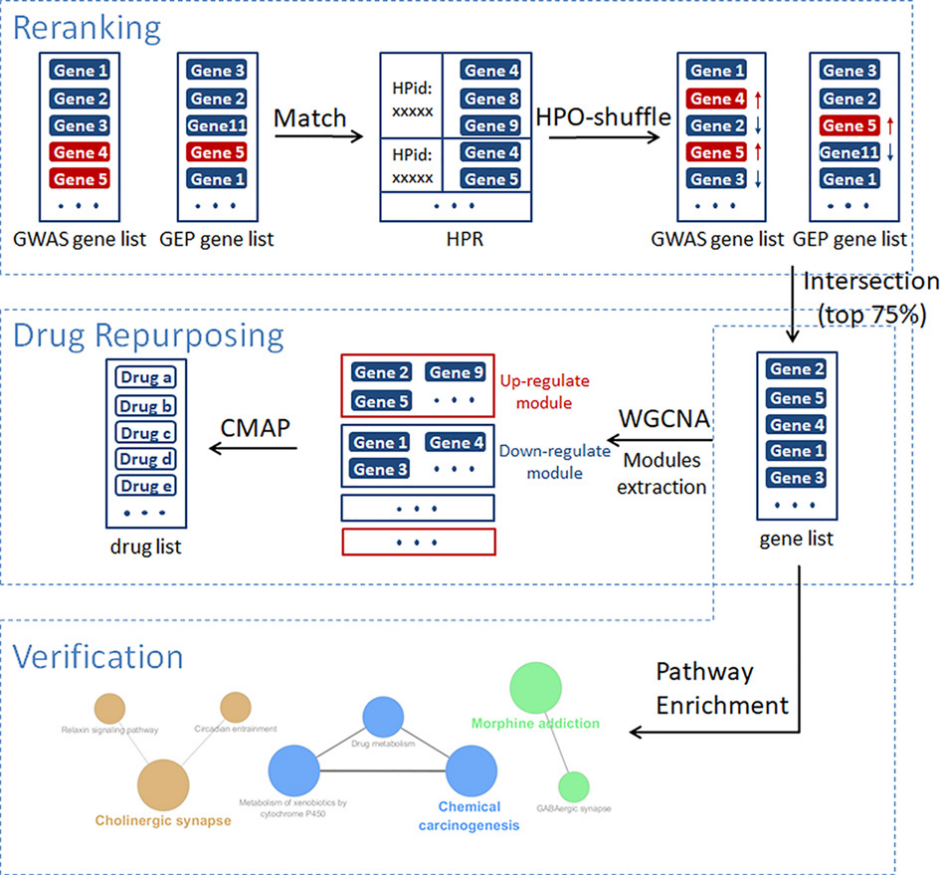
The pipeline of the proposed drug repurposing strategy The first step is the reranking process, and the second step is for drug repurposing, followed with the third step, verification.

The pipeline of this drug repurposing strategy is shown in Figure 1. Two of the original gene ranking lists were obtained from GWAS and GEP experiments, respectively. After matching to the epilepsy related HPO terms, genes were reranked according to the number of HPO terms that the gene matched, as indicated in the HPO-Shuffle algorithm. The genes that matched more epilepsy related HPO terms were assigned more scores and the top of the genes in the two reranked lists were considered to be more related to epilepsy. Afterward, the two reranked lists were merged as the final gene ranking list by taking the intersection of the top 75%. Subsequently, by using WGCNA, genes were clustered into different modules that intrinsically corresponded to different biological functions. The relation between module gene sets and sample traits was inferred by correlation between gene expression and sample phenotype. Moreover, the top five modules showing the strongest association with sample epilepsy status (affected or not) were considered in the subsequent CMap analysis. The genes were grouped based on the association direction and input into CMap platform, so as to obtain the list of potential epilepsy drugs. Eventually, pathway enrichment was performed to verify the significant relevance between the gene lists and the target disease, epilepsy.

**[Drug repurposing pipeline]:**

Step 1: Derive a canine gene ranking by GWAS analysis.
Step 2: Derive a parallel gene ranking from GEP microrray analysis.
Step 3: Filter and rerank the gene list by enrichment analysis of relevant HPO terms, so as to widen the selection range of GWAS genes with sufficient significance. Here, HPR repository is regarded as linkage repository that bridge the gene, phenotypes and observed disease.
Step 4: Evaluate the reranked gene list by prior knowledge of druggability info.
Step 5: Use WGNCA analysis to calculate the modular gene set.
Step 6: Use CMap analysis to obtain the potential antiepilepsy drug list with respect to the targeted module gene set.

## Result

### Evaluation of HPO-Shuffle in Relevant Set A

To evaluate the reliability of relevant gene filtering results by traditional gene association study, experiments of GWAS and GEP association studies were carried on separately.

In the GWAS data, there were 779 genes in the test set, and 1327 in the GEP data. To test the effect of the HPO-Shuffle, rank change of genes in both Relevant Set A and Key Set B were examined.

**Figure 2:**
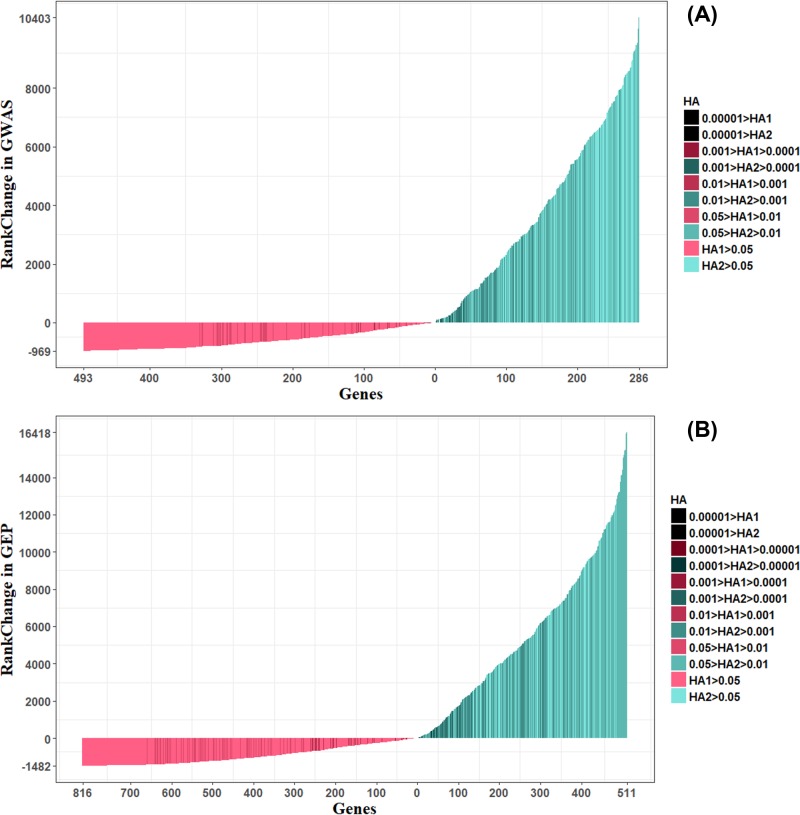
Rank change of Relevant Set A genes in GWAS lists after HPO-Shuffle The abscissa represents the number of genes, while the ordinate represents the ranking change after HPO-Shuffle. Each vertical line represents a gene, whose color indicates that the rise or fall of ranking and brightness indicates the magnitude of the HA value. (**A**) Rank change of Relevant Set A genes in GWAS list. (**B**) Rank change of Relevant Set A genes in GEP list.

First, the rank changes of genes in Relevant Set A were visualized in Figure 2 to show the general improvement of gene ranks after using HPO-Shuffle.

As shown in Figure 2, the *X*-axis corresponds to the number of genes, and the *Y*-axis corresponds to the ranking change after HPO-Shuffle. A red vertical line indicates decrease in gene ranking after HPO-Shuffle, while a green line indicates increase. In GWAS gene set, the rankings of 493 genes decreased and 286 genes increased, while in GEP gene set 815 genes decreased and 511 genes increased. The greatest decrease was 969 in GWAS gene set, and 1482 in GEP gene set. The greatest increase was 10,403 in GWAS gene set, and 16,418 in GEP gene set. The range of gene ranking increase was substantially greater than the range of gene ranking decrease, which suggested that the HPO-Shuffle significantly elevated the rank of genes closely relevant to epilepsy by considering their related phenotype, and the rank of genes that matched less HPO terms mostly did not decrease drastically.

The depth of shade in the line represents the *P*-value for the corresponding gene, i.e. the darker the line, the smaller the *P*-value of the corresponding gene. As shown in Figure 2, most of the ranking-increase genes had smaller *P*-values than ranking-decrease genes. In addition, the genes whose ranking sharply declined were generally with greater *P*-values. The Figure 2 indicates that HPO-Shuffle increased the genes that were more likely to be related to epilepsy, which makes the gene ranking more reliable. With the null hypothesis that ‘there is not significance difference between the rank increase and decrease tendency among genes after HPO shuffle’, Wilcoxon rank sum tests on the GWAS gene set and the GEP gene set were performed, respectively. The *P*-values of the two Wilcoxon rank sum tests were both 2*.*2 × 10*^−^*^16^, which strongly rejected the null hypothesis and suggested that the HPO shuffle increase the rank of Relevant Set A genes.

### Evaluation of HPO-Shuffle in Key Set B

**Figure 3:**
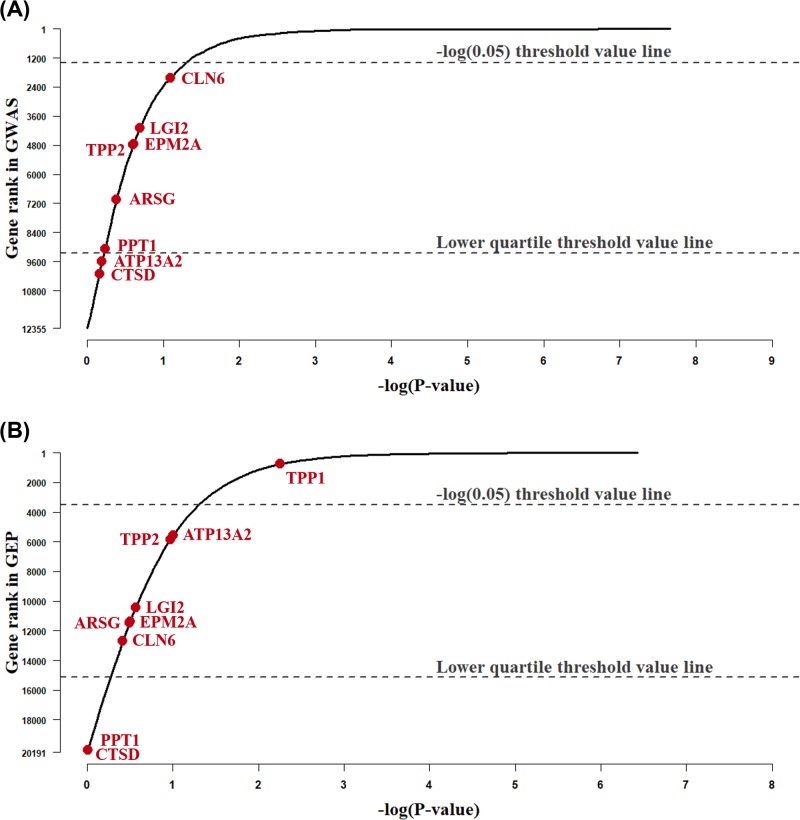
Drawback of single GWAS or GEP experiment in identifying key genes in Key Set B

Furthermore, the rank changes of genes in Key Set B were introduced in this section along with the description of drawback of the single GWAS or GEP analyses, where Figure 3 illustrates the awkward ranks of 8 key genes in Key Set B in the original GWAS and GEP analyses.

Here, genes were ordered and plotted in the curve, where those eight genes were marked in red. The *Y*-axis in Figure 3 denoted the gene rank with a descending order, and the *X*-axis referred to log *P* where *P*-value is the original *P*-value in GWAS or GEP analysis. In order to evaluate the rank of known associated genes, two horizontal lines were represented in both sub-figures as well. One was the −log(0*.*05) threshold value line, and the line intersected with the *Y*-axis at the the 1397th gene in GWAS and 3525th in GEP, either of which was the last one passed the conventional significance threshold (0.05) in GWAS or GEP analysis. Another horizontal line referred to the lower quartile, i.e. the top 75% genes were distributed above the lower quartile lines.

The result in Figure 3 clearly showed that neither GWAS nor GEP analysis achieved an effective identification of known associated genes. Both of the traditional methods failed to identify sufficient amount of key associated genes. As shown in Figure 3, there is only one key gene, TPP1, that was successfully retrieved in the GEP analysis. The deficiency of the key gene identification in traditional analyses warranted an enhancement in relevant gene prioritization.

By using HPO-Shuffle reranking algorithm, the GWAS and GEP gene list were updated, and the full results were listed in Appendix Table A2: Full result of GWAS and Appendix Table A3: Full result of GEP.

**Figure 4:**
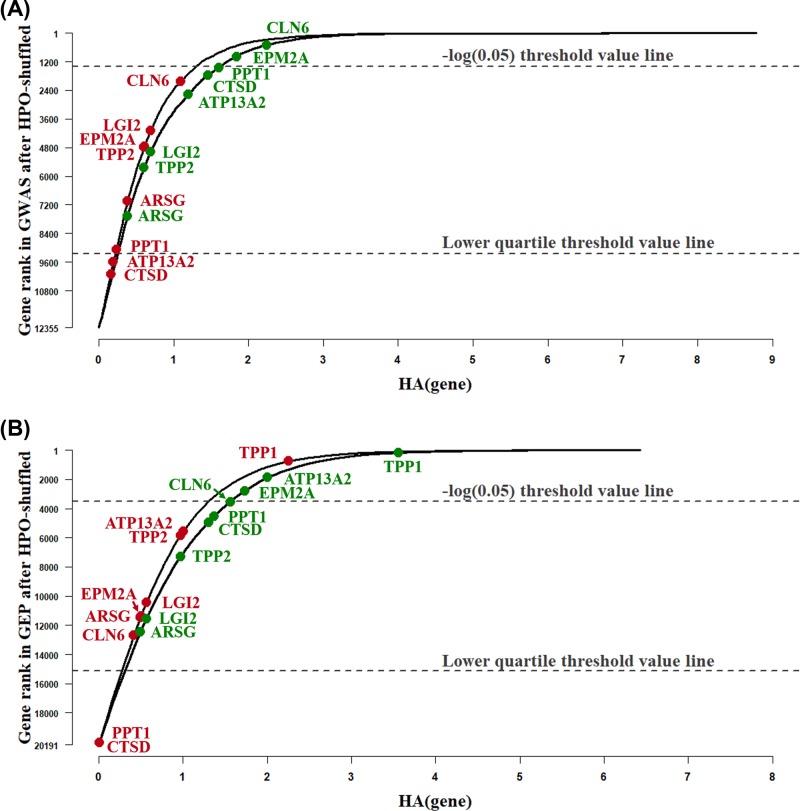
Rank change of Key Set B genes in GWAS and GEP lists after HPO-Shuffle

Based on this updated gene list, drug repurposing was due to be carried on, thus it was necessary to evaluate the robustness of the gene rank updating algorithm. In order to show the improvement of the updated gene list, the same gene set with reported epilepsy associated genes were observed both in the previous GWAS/GEP gene list and the updated reranked list, as shown in Figure 4 and Table 2.

**Table 2:** Comparison of reranked GWAS order for reported canine epilepsy genes

Breed	Gene mutated	Rank order in GWAS	Reranked order	Rank change
**Gene prioritization method: GWAS**				
American Bulldog	CTSD [27]	10,126	1766	(+8360) ↗
Dachshund	PPT1 [29]	9067	1464	(+7603) ↗
Tibetan Terrier	ATP13A2 [32]	9593	2575	(+7018) ↗
Miniature wirehaired Dachshund	EPM2B [26] (EPM2A)	4740	933	(+3747) ↗
Australian shepherd	CLN6 [31]	2030	499	(+1531) ↗
American Staffordshire terrier	ARSG [30]	7052	7677	(-625) ↘
Dachshund	TPP1 [28] (TPP2)	4796	5654	(-858) ↘
Lagotto Romagnolo	LGI2 [33]	4093	4989	(-895)↘
		-/12,355	-/12,355	

Breed	Gene mutated	Rank order in GEP	Re-ranked order	Rank change
**Gene prioritization method: GEP**				
Dachshund	PPT1 [29]	20,056	4547	(+15,509) ↗
American Bulldog	CTSD [27]	20,177	4943	(+15,234) ↗
Australian shepherd	CLN6 [31]	12,707	3536	(+9171) ↗
Miniature wirehaired Dachshund	EPM2B [26] (EPM2A)	11,389	2825	(+8564) ↗
Tibetan Terrier	ATP13A2 [32]	5582	1890	(+3692) ↗
Dachshund	TPP1 [28]	766	186	(+580) ↗
American Staffordshire terrier	ARSG [30]	11,488	12,474	(-986) ↘
Lagotto Romagnolo	LGI2 [33]	10,446	11,548	(-1102) ↘
		-/20,191	-/20,191	

The results shown in Figure 4 were under comparison with Figure 3, showing the rank changes of known epilepsy related genes. The upper horizontal dash line corresponded to the genes with a original *P*-value of 0.05. Meanwhile, the lower horizontal dash line corresponded to the gene which ranked at the topmost 75% ranking position. The red and green points referred to the ranking position of the eight known reported genes before and after HPO-Shuffle re-ranking strategy. Straightforward observation showed that most green points were clustered in the higher order area, compared with the red points. The visualization results in the Figure 4 were consistent with that in Table 2.

By offering more detailed rank changes, the results in Table 2 list worked well for the known genes. For example, CTSD was a known gene for epilepsy [27], whose ranking order moved upward from 10,126th to 1766th, while PPT1 [29] increased its ranking from 9067th to 1464th. For the 8 genes in the Key Set B, five of them had rank order dramatically increased than before. Furthermore, all of the associated genes moved upward and located above the lower quartile threshold value line.

Since the observed genes were all reported as related to epilepsy and seizures, the variation of rank result showed that the new ranking list achieved a reliable enhancement in gene prioritization.

### KEGG pathway enrichment for the intersection of the filtered gene sets

After selecting the first 75% genes of the reranked GWAS and GEP lists, the intersection contained 5639 genes. The intersection genes were obtained by integrating three sets of data: GWAS data, GEP data and HPO data.

**Figure 5:**
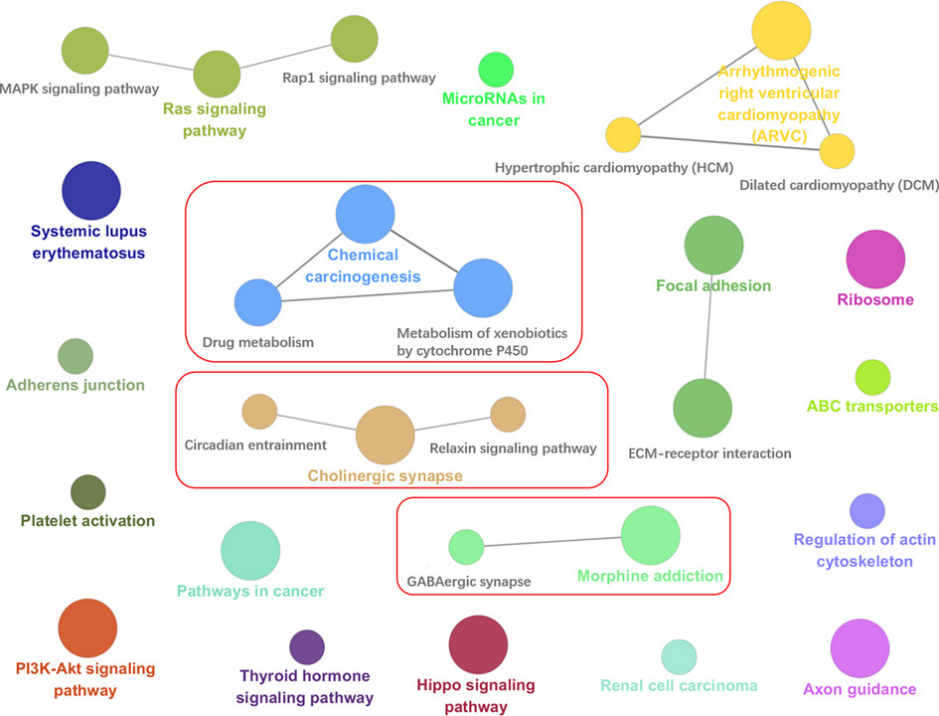
Result of ClueGO KEGG pathway enrichment

For observing the relevance of these filtered genes with epilepsy, we used ClueGO [41], a Cytoscape [42] plug-in, to perform KEGG enrichment on the intersection of the gene sets. In addition, ClueGO is capable of comparing clusters of genes and visualizing the functional differences [41]. ClueGO uses kappa statistics to link the terms in the KEGG pathway enrichment network, and the size of the nodes reflects the enrichment significance of the terms. We retained all KEGG pathways with *P*-values less than 0.05. KEGG pathway enrichment results in Figure 5 showed that most pathways were associated with epilepsy or the central nervous system. For example, the Cholinergic synapse pathway, acetylcholine (ACh) which is a neurotransmitter widely distributed in the central nervous system (CNS) and is closely related to epilepsy. In the KEGG database, most of diseases related to this pathway are in the same category with epilepsy. For instance, Morphine addiction pathway and GABAergic synapse pathway reflect the importance of GABA in the treatment of epilepsy. Guanidine-aminobutyric acid (GABA) modulator is an inhibitory transmitter of the central nervous system that promotes the influx of chloride ions into cells to make the hyperpolarization of the membrane more stable. Morphine is reported to indirectly activate VTA dopamine neurons by reducing GABAergic neuron-mediated inhibitory synaptic transmission. It is known that compounds which increase GABA content or prolong or increase sensitivity are regarded to have antiepileptic effects. In addition, the pathway named metabolism of xenobiotics by cytochrome P450 is critical in the metabolism of antiepileptic drugs. Cytochrome P450 (CYP) enzyme system is mainly found in liver microsomes and consists of three parts: heme protein (P450), flavin protein and phosphatidylcholine. CYP enzyme, as the first phase enzyme of drug metabolism, plays an important role in the metabolic rate of drugs, and closely relates to drug elimination rate. Most AEDs are metabolized by a variety of CYP isozymes.

### Module extraction of selected genes by WGCNA

The information contained in the microarray experiment is much richer than a list of differently expressed genes. It can be further exploited by considering the relationships between measured transcripts. To further discover their potential values, probes data of the intersection genes are used to construct weighted gene coexpression network by WGCNA [43]. It is predicted that the genes in the same module also shared the similar biological function. Instead of relating thousands of genes to the sample trait, WGCNA focused on relating vital traits and clusters of highly interconnected genes, which formed modules based on relevance score.

In our research, WGCNA was utilized to assess the pair-wise correlations between gene expression profiles and identify dozens of gene modules from the expression data of 5639 genes by hierarchical clustering. The basic statistics of module partition was shown in Table 3. There are five modules with *P*-value reached significance if the threshold was set to 0.05. In the meantime, the module scores obtained by WGCNA exceeded 0.5, which was the significance level for module clustering.

**Table 3:** Statistics summary for modules in WGCNA

Module name	Gene count	*P*-value	Module score
Light yellow	103	1e*^−^*^6^	0.86
Dark grey	88	0.008	0.58
Saddle brown	76	0.01	0.55
Purple	153	0.02	0.5
Cyan	127	0.03	0.5

**Figure 6:**
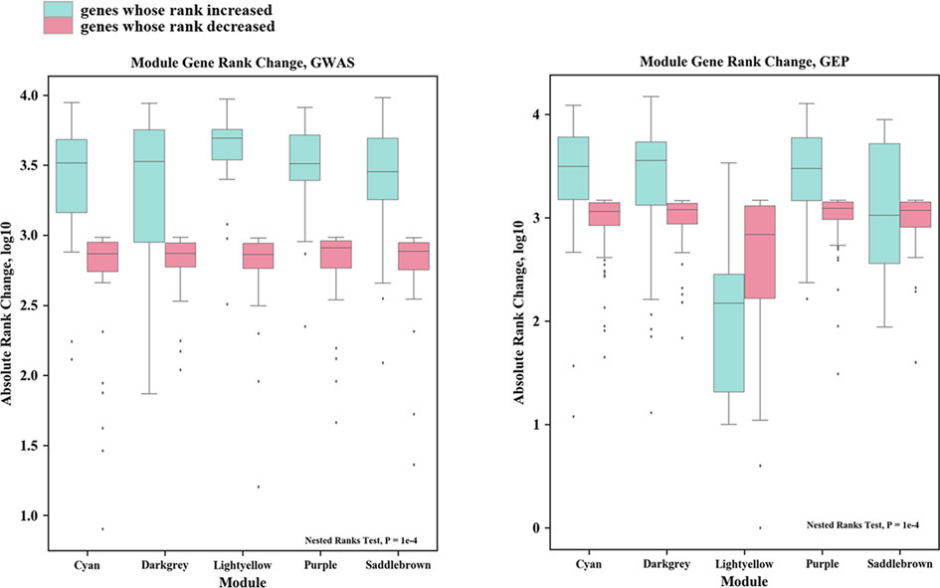
Rank optimization before and after HPO-Shuffle The green bar refers to gene whose rank increased, and the red bar refers to gene whose rank decreased.

To assess the reliability of WGCNA module partition result, rank optimization of module genes by HPO-Shuffle was evaluated. As shown in Figure 6, the green boxes correspond to log10 transformed absolute rank changes in genes whose rank increased after HPO-Shuffle, and red boxes genes whose rank decreased. Outliers shown as dark points. The absolute rank change of rank-increased genes was clearly larger compared to rank-decreased genes for all the included modules in the GWAS setting, and for most of the modules in the GEP setting. Further statistical investigation using nested ranks test, a mixed-effects extension of Wilcoxon test, reached statistical significance of 1e*^−^*^4^ for the difference in absolute rank change between rank-increased and rank-decreased module genes, in GWAS and GEP settings both. Being well suited in expectation, a good body of genes in the core modules obtained a significant rank increase, which laid rational basis for the following associated gene set selection.

## Discussion

### Hypergeometric test for the significance of phenotypic association of gene list with HPO-Shuffle

Top 75% of the GWAS list and GEP list were intersected to obtained a more reliable gene list. With a list of known epilepsy genes recorded in databases, a hypergeometrics test was performed to examine the enrichment specificity. The number of human genes is 19,238, while the epilepsy genes list contains 1460 genes. As showed in the Table 4, GWAS gene list contains 9266 genes, and 666 of them are in the epilepsy genes list. About 15,144 genes in GEP list which includes 1075 known epilepsy genes. After intersection, the total genes reduced to 5639 while the known epilepsy gene number is 627 which only differed by 39 genes from GWAS. Therefore, among the results, intersection got the best *P*-value, which was 3.4556E-31.

**Table 4:** Significance test for phenotypic association of HPO-Shuffle genes

	Total gene	Known epilepsy gene	*P*-value
**GWAS**	9266(75%)	666/1460	0.9773
**GEP**	15,144(75%)	1075/1460	0.9999
**Intersection**	5639	627	3.4556E-31

The success of this research relied heavily on the effectiveness of the HPO reranking algorithm. The results in the above hyper-geometric test partly showed the robustness of the re-ranked gene set.

### Evidence curation of the potential drug list

Three modules with *P*-values below 0.01 in Table 3, i.e. light yellow with 103 genes, dark gray with 88 genes, saddle brown with 76 genes, were extracted as significant WGCNA modules. Up-regulated and down-regulated expressed genes in the above three modules were submitted to the CMap database, which consisted of gene expression profiles of different cell lines affected by different interferences like small molecule compounds, overexpressed genes and gene knockouts.

**Figure 7:**
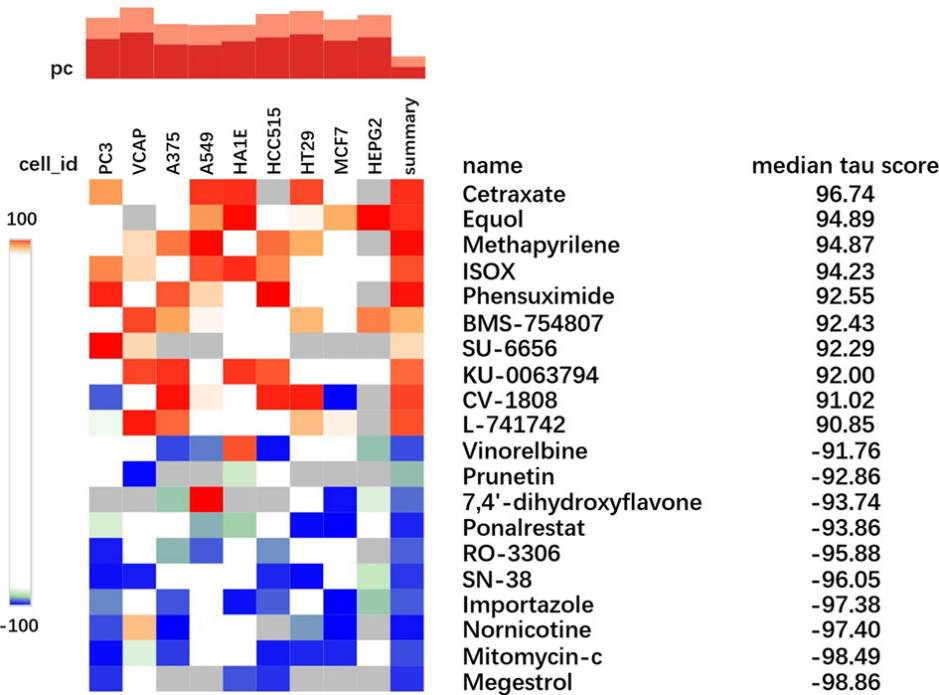
Top 20 compounds in the Connectivity map results The extreme blue color refers to minus 100 of *τ* value, and the extreme red color refers to +100 of *τ*.

By comparing the reference data, a list of cell expression profiles with high similarity and compounds that acts on cells were obtained. Top 20 compounds in the Connectivity map results was shown in Figure 7. CMap score Tau (*τ* ) is to compare an observed enrichment score to all other chemical perturbations, such as in CMap-L1000v1 [40]. A tau of 96.74 indicates that only 3.26% of reference chemical perturbations showed stronger connectivity to the queried gene sets so as to show in a reference database. This shows that Cetraxate is strongly relevant to the chemical perturbations upon the chosen get sets.

Each column of the heat map corresponds to a different cell line i.e. PC3, VCAP, A375, A549, HA1E, HCC515, HT29, MCF7 and HEPG2. These cell lines derived from cancerous tissues from different parts of humans are commonly used in experiments and the detail is shown in Table 5. Positive scores indicated that the up-regulation and down-regulation genes were similar to the reference gene expression profiles; negative scores indicated the opposite.

**Table 5:** Nine cancerous tissue cell lines used for CMap analysis

Cell Line	Culture properties	Description
A375	Adherent	Human malignant melanoma
A549	Adherent	Human non-small cell lung carcinoma
HCC515	Adherent	Human non-small cell lung adenocarcinoma
HEPG2	Adherent	Human hepatocellular carcinoma cell line
MCF7	Adherent	Human breast adenocarcinoma
PC3	Adherent	Human prostate adenocarcinoma
VCAP	Adherent	Human metastatic prostate cancer
HT29	Adherent	Human colorectal adenocarcinoma
HA1E	Adherent	Human kidney epithelial immortalized

From CMap experiments, all the high scoring compounds were associated with epilepsy in the CMap list. For example, compounds with higher absolute values (Cetraxate, equol, methapyrilene, ISOX, mitomycin-c and megestrol) were reported to have possibility to induce epilepsy. Especially megestrol, which was ranked as the last in the negative score, was specifically prohibited to patients with a history of epilepsy from taking it. The full table with additional description was shown in Table 6.

- **Reported drugs related to epilepsy with direct evidence.** Megestrol, Mitomycin-c, Cetraxate, ISOX, 7,4′-dihydroxyflavone, Phensuximide.
  1. Megestrol: Megestrol is a progestogen drug that can be used as an appetite stimulant to treat digestive syndrome. It is also used to treat breast and endometrial cancer and has been used for birth control. The corresponding drug instructions all suggest that patients with epilepsy, migraine and depression should be used with caution.
  2. Mitomycin-c: Mitomycin-c is an antitumor drug isolated from the culture medium of actinomycetes and is effective against various solid tumors. It is one of the commonly used periodic non-specific drugs. After activation by a reductase in a cell, the DNA of the cell can be depolymerized while blocking DNA replication, thereby inhibiting tumor cell division. At high concentrations, Mitomycin also inhibits the synthesis of RNA and protein. It is warned that ingestion of mitomycin may result in sudden onset of seizures or loss of consciousness in PubChem database (https://pubchem.ncbi.nlm.nih.gov/compound/mitomycin_C#section=Top).
  3. Cetraxate: Cetraxate can improve gastric microcirculation, enhance the resistance of gastric mucosa, increase the synthesis of prostaglandin E2 and prostaglandin I2 in gastric mucosa, inhibit the secretion and activation of gastric acid and pepsin, increase the secretion of gastric mucus and promote ulcer healing. Cetraxate also fights against smooth muscle spasms. In the TOXNET database, this compound has been reported to cause convulsions or has an effect on seizures threshold.(https://chem.nlm.nih.gov/chemidplus/rn/27724-96-5)
  4. ISOX: Isoxsuprine is a drug that blocks alpha-receptors and agonizes beta-receptors. It also directly relaxes vascular smooth muscle and uterine smooth muscle. Therefore, it can relax the cerebral blood vessels, skeletal muscle blood vessels and skin blood vessels. Clinically used for the treatment of cerebrovascular and peripheral vasospasm. Isoxsuprine hydrochloride has a record in the TOXNET database indicating that it may cause seizures (https://toxnet.nlm.nih.gov/cgi-bin/sis/search/a?dbs+hsdb:@term+@DOCNO+3106).
  5. 7,4′-dihydroxyflavone: 7,4′-dihydroxyflavone, like many other flavonoids, is an opioid receptor antagonist. Specifically, it acts as an antagonist of the μ-opioid receptor, and the κ-and delta-opioid receptors also have relatively low affinity. 7,8-dihydroxyflavone, which is a isomer of 7,4′-dihydroxyflavone, can be used to treat a variety of human diseases associated with the brain’s nervous system [44] and its derivatives are beneficial to the nervous system and exhibit an effective antidepressant effect [45]. In addition, 5,7-dihydroxy-4′-methoxyflavone is a drug used to prevent or reduce the severity of seizures according to ChEBI database (https://www.ebi.ac.uk/chebi/searchId.do?chebiId=CHEBI:15335).
  6. Phensuximide: Phensuximide is a common antiepileptic drug that acts similarly to ethosuximide but is weaker. Ethosuximide is recommended as the preferred initial therapy for childhood absence epilepsy in a recent study [46], which is consistent with our samples’ trait (idiopathic absence epilepsy). The advantages of ethosuxamine are safe, effective, no sedative effect and long elimination half-life.
- **Reported drugs related to epilepsy with indirect evidence.** Nornicotine, SN-38, RO-3306, Equol, Methapyrilene, Prunetin, BMS-754807, SU-6656, KU-0063794, Vinorel-bine, CV-1808, L-741742.
  1. Nornicotine: Nornicotine is an acetylcholine receptor agonist. Neuronal AChRs are sites of action for nicotine and are potential targets for the treatment of schizophrenia, Alzheimer’s disease, epilepsy and other human neurological diseases [47].
  2. SN-38: SN-38 is the active metabolite of irinotecan which is a topoisomerase I (Top1) inhibitor. A research pointed that reducing the transcription of Top1 gene is helpful to suppress seizure. Thus, Top1 inhibitors are considered to be the possible antiepileptic drugs.
  3. RO-3306: RO-3306 is a potent and selective inhibitor of CDK1 (cyclin-dependent kinase 1), associated with cell cycle and mitosis. CDK1 is thought to be the ‘main switch’ in cell division, which maintains the mitotic state of cells. Glioneuronal lesions caused by focal cortical dysplasias (FCDs), which are frequently encountered in biopsy specimens of patients with pharmacoresistant focal epilepsy, may be related to the impaired expression of CDK1 in FCD [48].
  4. Equol: Equol is a metabolite of soy, an estrogen receptor agonist and an important isoflavone in humans. A study in 2014 investigated potential associations between the consumption of soy-based infant formula and epilepsy, and hypothesized that soy phytoestrogens interfere with metabotropic glutamate receptor signaling through an estrogen receptor-dependent mechanism, which results in elevated production of key synaptic proteins and decreased seizure threshold in genetically susceptible individuals [49].
  5. Methapyrilene: Methapyrilene is an antihistamine and anticholinergic of the pyridine chemical class. After being shown to be a potent carcinogen, mthapyriline drug products have been voluntarily withdrawn by Manufacturers from the market in 1979. There are also many warnings about Methapyrilene-induced seizures in the TOXNET database.(https://toxnet.nlm.nih.gov/cgi-bin/sis/search/a?dbs+hsdb:@term+@DOCNO+4163)
  6. Prunetin: Prunetin is an O-methylated isoflavone and a inhibitor of breast cancer resistance protein (BCRP). It has the function of inhibiting tumors. BCRP may play a crucial role in the development of drug resistance in MTLE and may serve as potential prognostic markers of drug resistant epilepsy. As it can be blocked by specific inhibitors, such inhibitors can therefore be used as adjunctive treatment for drug resistant epilepsy. [50]
  7. BMS-754807: BMS-754807 is the inhibitor of IGF-1R and is used to tumor treatment. There is not a report about this drug with epilepsy. However a known mechanism of epilepsy is that high level of IGF-1 could promote the development of seizure activity by enhancing the excitability of hippocampal neurons. Therefore, the inhibitor of IGF-1R may help to attenuate seizure activity (http://www.clinsci.org/content/129/12/1047).
  8. SU-6656: SU-6656 is a highly active Src kinases inhibitor. Src kinases are involved in NMDAR (N-methyl-D-aspartate subtype of glutamate receptor) phosphorylation during status epilepticus and were described as ‘a hub for NMDAR regulation’. Some forms of epilepsy are thought to depend on upregulation of NMDAR function [51]. Strong inhibitory activity on the family of the Src kinases may inhibit the spread of the epileptiform activity to the contra lateral hippocampus. [52]
  9. KU-0063794: KU-0063794 is a novel potent and highly specific small-molecule inhibitor of the mTOR protein kinase and inhibits both mTORC1 and mTORC2 *in vitro* and *in vivo* [53]. It has been demonstrated that hyperactivation of mTOR signaling is a common occurrence in epilepsy. And mTOR inhibition is an exciting potential antiseizure and antiepileptogenic strategy [54].
  10. Vinorelbine: Vinorelbine is tubulin inhibitor. Microtubule inhibitor and mitotic inhibitor, microfilament proteins, tubulin, cytoskeleton etc. were also included in the results of KEGG enrichment. Tubulin Beta 3 Class III (TUBB3) is the most dynamic Beta-tubulin isoform expressed in neurons, and is highly expressed in the central nervous system and TUBB3 expression was up-regulated in human and rat epileptic tissue [55]. The administration of microtubule-modulating agent is able to attenuate the progression of chronic epilepsy.
  11. CV-1808: CV-1808 is an adenosine receptor agonist. Adenosine, as the brain’s endogenous anticonvulsant, is considered to be responsible for seizure arrest and postictal refractoriness [56]. Adenosine receptor agonist is able to inhibit drug-resistant seizures and is a new epilepsy treatment based on adenosine.
  12. L-741742: L-741742 is an effective and highly selective dopamine D4 receptor (D4R) antagonist. Dopamine D4 receptor is strongly linked to neuropsychiatric disorders [57]. Experimental records show that the frequency of spontaneous synaptic activity and the frequency and duration of paroxysmal discharges induced by epileptogenic agents were increased in D4R-deficient mice compared with wild-type mice. [58]
- **Unreported drugs.** Importazole, Pnalrestat.
  1. Importazole: Importazole is a small molecule inhibitor of the transport receptor importin-β, which carries cargoes into the nucleus during interphase. [59]
  2. Pnalrestat: Poonastat is a chemical substance used in antidiabetic drugs for aldose re-ductase inhibitors. The aldose reductase inhibitors has encouraging results from clinical trials of diabetic neuropathy. [60]

**Table 6:** Top 20 compounds in the connectivity map results

Name	Median *τ* score	Evidence	Description
Megestrol	-98.86	√ √	Progesterone receptor agonist, DNA inhibitor, HegG2 inhibitor
Mitomycin-*c*	-98.49	√ √	DNA Alkylating drug, DNA inhibitor, DNA synthesis inhibitor
Nornicotine	-97.40	√	Acetylcholine receptor agonist
Importazole	-97.38	NA	Importin-transport receptor inhibitor
Cetraxate	96.74	√ √	Gastrin inhibitor, mucus protecting agent
SN-38	-96.05	√	Topoisomerase inhibitor
RO-3306	-95.88	√	CDK inhibitor
Equol	94.89	√	Estrogen receptor agonist
Methapyrilene	94.87	√	Histamine receptor antagonist
ISOX	94.23	√ √	HDAC inhibitor
Ponalrestat	-93.86	NA	Aldose reductase inhibitor, reductase inhibitor
7,4′-dihydroxyflavone	-93.74	√ √	Opioid receptor antagonist
Prunetin	-92.86	√	Breast cancer resistance protein inhibitor
Phensuximide	92.55	√ √	Anticonvulsant
BMS-754807	92.43	√	Insulin growth factor receptor inhibitor
SU-6656	92.29	√	Src inhibitor
KU-0063794	92.00	√	mTOR inhibitor
Vinorelbine	-91.76	√	Tubulin inhibitor, apoptosis stimulant, microtubule inhibitor, mitosis inhibitor
CV-1808	91.02	√	Adenosine receptor agonist
L-741742	90.85	√	Dopamine receptor antagonist

√ √: Reported drugs related to epilepsy with direct evidence, √: Reported drugs related to epilepsy with indirect evidence,

NA: Unreported drugs.

### Possibility of the large-scale drug repurposing by using this strategy

In the case study, 20 compounds were eventually filtered out through HPO-Shuffle based strategy. The literature research collected abundant evidence showing that 6 out of 20 compounds showed strong drug efficacy for epilepsy, while 12 out of 20 hinted indirect link to epilepsy cure, and henceforth provided potential drug efficacy.

Several AEDs of the older generation approved for humans have been proved to be unsuitable for the treatment of canine epilepsy as most have a short elimination half-life, which is not convenient to dose by owners, including phenytoin, carbamazepine, valproic acid and ethosuximide. Some are even toxic in dogs such as lamotrigine and vigabatrin [61]. Therefore, the key to discovering new AEDs is to find drugs that are non-toxic to dogs and have a long elimination half-life *in vivo*.

Some experiments have shown that after receiving phensuximide at 50 and 100 mg/kg orally (5 days a week, for 6 months to 1 year), dogs have no abnormalities shown on hematological and biochemical analyses of the blood and on gross and microscopic examinations of various organs and tissues [62].

Research on the metabolism half-life of phensuximide in dogs is still rare. However, some experiments on human patients have shown that methsuximide, a methylated homolog of phensuximide, has excellent performance in some patients because the half-life of its desmethyl metabolites is significantly prolonged. Therefore, we speculate that phensuximide and its chemical modifications potentially contain better canine antiepileptic drugs [63].

The research, along with the case study, proposed a promising gene prioritization and association for the purpose of drug repurposing in canine epilepsy.

## Data Availability Statement

Data available on request from the authors. The data that support the findings of this study are available from the corresponding author upon reasonable request.

## Supporting information

**Supplementary Table S1:** Supplementary Tables
